# Efficacy and safety of praziquantel 40 mg/kg in preschool-aged and school-aged children: a meta-analysis

**DOI:** 10.1186/s13071-016-1958-7

**Published:** 2017-01-26

**Authors:** Julien Zwang, Piero Olliaro

**Affiliations:** 1 Independent researcher, Bangkok, Thailand; 2 UNICEF/UNDP/World Bank/WHO Special Programme for Research & Training in Tropical Diseases (TDR), Geneva, Switzerland; 3 Centre for Tropical Medicine and Global Health, Nuffield Department of Medicine, University of Oxford, Oxford, UK

**Keywords:** Schistosomiasis, Praziquantel, Preschool-aged children, School-aged children, Efficacy, Safety

## Abstract

**Background:**

Children carry most of the schistosomiasis burden. While school-aged children are the principal target group of preventive chemotherapy with praziquantel, limited information on efficacy and safety exists for preschool-aged children.

**Methods:**

Here, we conducted a meta-analysis of clinical trials of praziquantel for treating children with any form of schistosomiasis. Efficacy was reported as cure rate (CR) and egg reduction rates (ERR); statistical corrections were applied based on methodological disparities across trials to derive the predicted geometrical mean ERR (pERRgm). Safety was reported as frequencies of adverse events.

**Results:**

Forty-seven comparative and non-comparative studies were identified, enrolling 15,549 children of whom 14,340 (92%) were assessed between 3 and 8 weeks post-treatment with praziquantel 40 mg/kg (the WHO-recommended treatment, *n* = 8,380, 56%) or comparators (*n* = 5,960, 44%). The median age was 10 years (range 1–19), 11% (*n* = 1,694) were preschool-aged. The CR and pERRgm with praziquantel 40 mg/kg were respectively: *S. haematobium*, 73.6% (95% CI: 63.5–81.40, 25 study arms) and 94.7% (95% CI: 92.7–96.4); *S. mansoni*, 76.4% (95% CI: 71.5–81.0, 34 arms) and 95.3% (95% CI: 94.2–96.2); *S. mansoni*/*S. haematobium*, 67.6% (95% CI: 54.1–80.7, 5 arms) and 93.4% (95% CI: 89.9–96.2); *S. japonicum*, 94.7% (95% CI: 92.2–98.0) and 98.7% (95% CI: 98.3–99.2).

Mixed-effect multivariate analysis found no significant difference between preschool- and school-aged children for CR or pERRgm in S. *haematobium* (*P* = 0.309 and *P* = 0.490, respectively) or S. *mansoni* (*P* = 0.982 and *P* = 0.895) after controlling for time of assessment, formulation, intensity of infection and detection method. Praziquantel was reportedly safe at all ages, with only mild reported adverse events which cleared rapidly after treatment.

**Conclusions:**

Praziquantel 40 mg/kg was effective at reducing infection intensity in all *Schistosoma* species without differences between preschool- and school-aged children. However, conclusions should be tempered because of the limited number of preschool-aged children enrolled, disparities in study procedures and limited information made available in publications, as well as the current imperfect test-of-cure. Also, although reportedly well-tolerated, safety was inconsistently assessed. Studies in target groups, individual-data meta-analysis and standardised methodologies are needed for more robust evidence-base.

**Electronic supplementary material:**

The online version of this article (doi:10.1186/s13071-016-1958-7) contains supplementary material, which is available to authorized users.

## Background

Children carry most of the schistosomiasis burden [1]. Schistosomiasis treatment and control relies largely upon preventive chemotherapy (PC) with praziquantel (PZQ) directed primarily at school-aged children living in schistosomiasis-endemic areas [2]. Preschool-aged children have traditionally been excluded from PC and treated on confirmation of infection [3], but are recognized as a vulnerable group [4–6]. Emphasis is now shifting from control to elimination of schistosomiasis [7], meaning that all the infected groups should be treated, including preschool-aged children (1–4 years-old), provided a suitable formulation is available. PZQ is registered for use in children from four years of age, and can be given routinely in PC programmes to children measuring 94 cm or more (recently extended to 60 cm [8]) but the absence of paediatric formulations is a practical limitation to having younger children treated routinely.

Praziquantel has been in use now for over three decades, and has been given to millions of people, mostly children - close to 40 million school-aged children in 2013 [9]. However, several unresolved questions still exist about this drug (reviewed in Stothard et al. [10]), including its pharmacokinetic and pharmacodynamic properties [11].

The World Health Organization (WHO) recommends using PZQ at 40 mg/kg, a recommendation generally supported by systematic reviews [12–14] and to express treatment outcome in terms of egg reduction rate (ERR) calculated as the difference of the arithmetic mean egg counts (preferred over geometric means) between pre- and post-treatment samples [3]. It is worth adding, as a note of caution, that we only have imprecise methods to detect *Schistosoma* infection that are based on the detection of eggs in excreta (faeces or urine) as opposed to adult worms, and that these methods lack sensitivity (for instance, the widely-used Kato-Katz method cannot detect less than 24 eggs per gram of stools). Therefore, the (temporary) reduction or cessation of egg-excretion should not be interpreted as being equivalent to killing of adult worms. This has profound implications both for the estimation of schistosomiasis cases and for the assessment of drug efficacy [15].

This meta-analysis was conducted to assess the efficacy and safety of PZQ in preschool- and school-aged children and whether these outcomes differ with subject’s age. This question is particularly relevant in view of the inclusion of preschool-aged children as a target of schistosomiasis control through PC, and whether any dose adjustment should be required.

## Methods

This is a meta-analysis of aggregated data; eligible studies were comparative and non-comparative clinical trials where PZQ had been given at any dose to preschool- and school-aged children and adolescents) for treating intestinal or urinary schistosomiasis, and where outcome was assessed within 2 months (3 to 8 weeks) post-treatment. The study was conducted according to the PRISMA guideline (Preferred Reporting Items for Systematic Reviews and Meta-Analyses) [16]. The PRISMA checklist was used to ensure inclusion of relevant information in the analysis (Additional file 1: Table S1).

Published studies were identified by the Cochrane collaboration through electronic searches from January 1, 1990, up to November 2015 of MEDLINE, EMBASE, LILACS, the Cochrane Infectious Diseases Group’s trials register and the Cochrane Central Register of Controlled Trials (CENTRAL) using the search term “praziquantel”, regardless of language (English, French, and Portuguese) and might have failed to identify articles published in other languages.

To qualify for inclusion, trials were to be on PZQ mono-therapy at any dosage and dosing regimen, using any formulation and brand, and could be either non-comparative or comparative (randomized controlled trial, quasi-randomised trials). The search identified 828 studies of PZQ in schistosomiasis in children, of which 47 had an assessment within 8 weeks (subjects treated with praziquantel, control treatment or placebo). A previous paper reported the results of a broader meta-analysis conducted up to 2012 including subjects of all ages [14].

Efficacy outcomes are reported as cure rate (CR) and egg reduction rate (ERR). CR, defined as the conversion from a positive test pre-treatment to a negative test up to 8 weeks post-treatment, is reported as provided in the articles on a per-protocol population. ERR, defined as the proportional reduction in the mean eggs per gram of faeces (intestinal schistosomiasis) or per ml of urine (urinary schistosomiasis) post-treatment *vs* pre-treatment is presented based on the arithmetic (ERRam) or geometric (ERRgm) mean ERR provided in the articles, respectively, 5 and 31 of the studies included in this review (11 studies did not report ERR). Since ERR was inconsistently assessed on all patients in the per-protocol population or only those who had countable eggs in their excreta post-treatment (‘uncured’), a logarithmic correlation between the CR and the ERRgm (log-transformed) was applied to correct the ERRgm results based on uncured patients and to help predict the ERRgm in studies that reported only the CR.

Tolerability is assessed by calculating the incidence of adverse events (AE) defined as any sign or symptom occurring after treatment, irrespective of whether that sign or symptom was present at baseline or not, of its severity and drug-event relationship. The mean incidence is presented for the PZQ 40 mg/kg treatment groups (all brands) excluding PZQ 40 mg/kg syrup, and Levo-PZQ 20 mg/kg.

The 95% confidence intervals (CI) for the mean CR, ERR, and AE are calculated using a bootstrap resampling method based on statistical bias between studies with a maximum of 1,000 replicates [17]. For randomized controlled trials assessing the efficacy (CR) and tolerability (AE) of PZQ *vs* other drugs, placebo, or comparing different PZQ dosing regimens, risk ratios (RR) with 95% CI, meta-analysis regression with random effect on the study/site is used and the pooled RR is presented using the DerSimonian & Laird procedure for random effects models [18]. Heterogeneity was expressed as I^2^ [19].

Children’s age groups were differently categorized whether the study was school-based (between 5 and 17 years of age) or community based including (i) preschool-aged (between 1 and 5 or 7 years) and (ii) school-aged (between 4 and 19 years) according to study definition. It should be noted that studies used categories which only partly conform with the WHO age classification for PC: preschool-aged 1–4-year-old; school-aged children 5–14-year-old.

Age-groups were inconsistently defined and generally broader in community-based studies; for example, children in nursery were enrolled as school-aged in a community based study [20]. As the studies had disparate age ranges, a median age (in year) was calculated from the reported range. A multivariate mixed-effect model was used to assess the age-dependency of CR and ERR with random effect on the site in an attempt to account for any potential statistical heterogeneity across studies while controlling for potential confounders such as: (i) endpoint of assessment (analysed as a continuous variable, in week); (ii) differences in diagnostic approaches (binary, single *vs* multiple smears); (iii) infection intensities before treatment, categorized as light (<100 egg per gram of faeces), moderate (100–399), and heavy (≥400) for *S. mansoni*, and categorized as light (<50 eggs/10 ml urine) and heavy (≥50) for *S. haematobium*; (iv) year of study.

Graphical displays for CR and ERR by age group were illustrated using forest plots [21]. Data were analysed using Stata v13 (Stata Corp.).

We also provide more detailed narrative summaries of the studies treating specifically preschool-aged children and those reporting results for narrower age-groups, where more direct comparisons are possible between more homogenous populations.

## Results

This analysis is based on 47 studies [20, 22–67] enrolling 15,549 children aged 6 months to 19 years (of whom 1,694 (11%) were preschool-aged children). Of these 14,340 were assessed for efficacy between 3 and 8 weeks after treatment (8% attrition, acceptable risk of attrition bias) (Table 1, Additional file 2: Table S2) of whom 11% (*n* = 1,506) were preschool-aged (5 studies) and 89% (*n* = 12,834) school-aged (42 studies), 48% (n = 6,897) infected with *S. mansoni*, 39% (*n* = 5,552) with *S. haematobium*, 8% (*n* = 1,196) with *S. japonicum*, and 5% (*n* = 695) co-infected with *S. mansoni/S. haematobium*. Fifty-eight percent (58%, *n* = 8,380) of the subjects were treated with PZQ 40 mg/kg (tablets, *n* = 8,037 or syrup, *n* = 343), 14% (*n* = 2,013) with other doses of PZQ, and 30% (*n* = 4,302) with other drugs or placebo. Outcomes were measured within 4 weeks post-treatment in 56% (*n* = 8,042) and within 4 to 8 weeks in 44% (*n* = 6,298) of the children. No study enrolled both preschool- and school-aged children.

**Table 1:** Participant and methodological characteristics of the studies included in the review

Species	Publication	Country	End-point (month)^a^	Median age (range, yrs)	Attrition				Egg count method
Study reference					Enrolled (*n*)	Assessed (*n*)	Assessed (%)	Risk of bias	
*S. haematobium*									
[27]	Borrmann et al. (2001)	Gabon	2	9 (5–13)	300	296	99	low	gm
[28]	Davis et al. (1979)	Zambia	1	12 (7–17)	151	151	100	low	gm
[29]	de Clercq et al. (2002)	Senegal	2	10.5 (7–14)	288	267	93	low	gm
[20]	Inyang-Etoh et al. (2009)	Nigeria	2	12 (4–20)	312	262	84	high	am
[30]	Keiser et al. (2010)	Ivory coast	1	12 (8–16)	83	83	100	low	gm
[31]	Keiser et al. (2014)	Ivory coast	1	11 (6–16)	78	71	91	low	gm + am
[32]	Latham et al. (1990)	Kenya	2	11 (7–15)	48	48	100	low	am
[33]	McMahon et al. (1979)	Tanzania	1	11 (7–15)	138	125	91	low	gm
[34]	Midzi et al. (2008)	Zimbabwe	2	11 (5–17)	675	624	92	low	na
[35]	N’goran et al. (2003)	Ivory coast	1	10 (5–15)	440	354	80	high	gm
[36]	Oyideran et al. (1981)	Nigeria	1	10 (7–13)	90	82	91	low	am
[37]	Ojurongbe et al. (2014)	Nigeria	1	10 (4–15)	245	245	100	low	gm
[38]	Ouldabdallahi et al. (2013)	Mauritania	1	15 (10–19)	151	151	100	low	na
[39]	Senghor et al. (2015)	Senegal		10 (5–15)	241	237	98	low	gm
[40]	Sissoko et al. (2009)	Mali	1	11 (6–15)	800	781	98	low	gm
[41]	Stete et al. (2012)	Ivory Coast	2	11 (7–15)	98	90	92	low	gm + am
[42]	Tchuente et al. (2004)	Cameroon	1	na, school-aged	674	515	76	high	gm
[43]	Wilkins et al. (1987)	Gambia	1	11 (5–17)	619	619		na	gm
*S. japonicum*									
[44]	Belizario et al. (2008)	Philippines	1	14.5 (10–19)	206	203	99	low	gm
[45]	Olliaro et al. (2011)	Philippines	1	9.5 (7–12)	203	200	99	low	gm
*S. haematobium*, *S. japonicum*, *S. mansoni*									
[46]	Olds et al. (1998)	Kenya, Phillipines, China	2	na, school-aged	1,540	1,540	100	low	gm
*S. mansoni*									
[47]	Barakat et al. (2011)	Egypt	2	12 (6–19)	588	588	100	high	gm
[48]	Barakat et al. (2015)	Egypt	2	10 (9–11)	268	194	72	low	gm
[49]	Berhe et al. (1999)	Ethiopia	2	11 (5–17)	611	541	89	high	gm
[50]	Botros et al. (2005)	Egypt	1, 2	14.5 (12–17)	144	103	72	high	gm
[51]	Degu et al. (2002)	Ethiopia	2	12 (10–14)	154	148	96	low	am
[52]	Erko et al. (2012)	Ethiopia	1		224	144	64	low	gm
[53]	Friis et al. (1988)	Botswana	2	na, school-aged	81	81	100	low	gm
[54]	Gryseels et al. (1987)	Burundi	2	12 (5–19)	630	630		na	gm
[55]	Guisse et al. (1997)	Senegal	1	10 (5–15)	130	130	100	low	gm
[56]	Massoud et al. (1984)	Egypt	1	na, school-aged	179	179	100	low	gm
[57]	Metwally et al. (1995)	Egypt	1	12 (8–16)	506	366	72	high	gm
[58]	Mohamed et al. (2009)	Sudan	1	12.5 (8–17)	102	92	90	low	gm
[25]	Nalugwa et al. (2015)	Uganda	1	Pre-school; (1–5)	714	696	97	high	gm
[24]	Navaratnam et al. (2012)	Uganda	1	Pre-school; 3 (1–5)	297	203	68	high	am
[59]	Obonyo et al. (2010)	Kenya	1	9.5 (7–12)	212	204	96	low	gm
[38]	Ouldabdallahi et al. (2013)	Tanzania	1	na, school-aged	271	244	90	low	gm
[60]	Selim et al. (2014)	Egypt	1	11.5 (6–15)	66	66	100		gm
[61]	Simonsen et al. (1990)	Ethiopia	1	9.5 (5–14)	265	206	78	high	gm
[26]	Sousa–Figueiredo et al. (2012)	Uganda	1	Pre-school; 4 (1–7)	369	305	83	high	am
[63]	Teesdale et al. (1984)	Malawi		12 (9–15)	69	69	100	low	am
[62]	Thiongo’o et al. (2002)	Kenya	2	11 (5–17)	1,018	1,018		na	gm
[64]	Utzinger et al. (2000 tmih	Ivory coast	1	10 (6–14)	253	194	77	high	gm
*S. mansoni* + *S. haematobium*									
[22]	Coulibaly et al. (2012)	Ivory Coast	1	Pre-school; (0.5–5)	53	53	100	high	gm
[65]	El Tayeb et al. (1988)	Sudan	1	9.5 (7–12)	111	111	100	low	na
[23]	Garba et al. (2013)	Niger	1	Pre-school; (2–5.5)	261	249	95	high	gm
[66]	Kardaman et al. (1985)	Sudan	2	9 (7–11)	220	211	96	low	na
[67]	Taylor et al. (1988)	Zimbabwe	1	12.5 (10–15)	373	283	76	high	gm

*Abbreviations: am* arithmetic mean, *gm* geometric mean, *na* not available

^a^1 month when assessment made at ≤ 1 month; 2 months is > 1 month and ≤ 2 months 5–8 weeks

The 1,694 pre-school-aged children were treated at five sites in Uganda, Niger and Ivory Coast with PZQ 40 mg/kg (tablet or syrup) or PZQ 2*40 mg/kg for *S. mansoni* and *S. haematobium*. The attrition at the one-month post-treatment assessment was 11%. Of the 1,506 children assessed, 12% (*n* = 179) were infected with *S. haematobium* and were either treated with PZQ 40 mg/kg tablet in a single-arm study (*n* = 18) [22] or with syrup only also in a single-arm study (*n* = 161) [23] and 88% (*n* = 1,327) were infected with *S. mansoni*: a two-arm study comparing crushed tablets and syrup, both given at 40 mg/kg (*n* = 203) [24]; a two-arm study [25] comparing PZQ tablets given as a single dose of 40 mg/kg (*n* = 357) to a double-dose of 2*40 mg/kg (*n* = 339); two single-arm studies [22, 26] with crushed tablets only (*n* = 369 and 35, respectively); and a single-arm study (with syrup only; *n* = 88) [23].

**Fig. 1:**
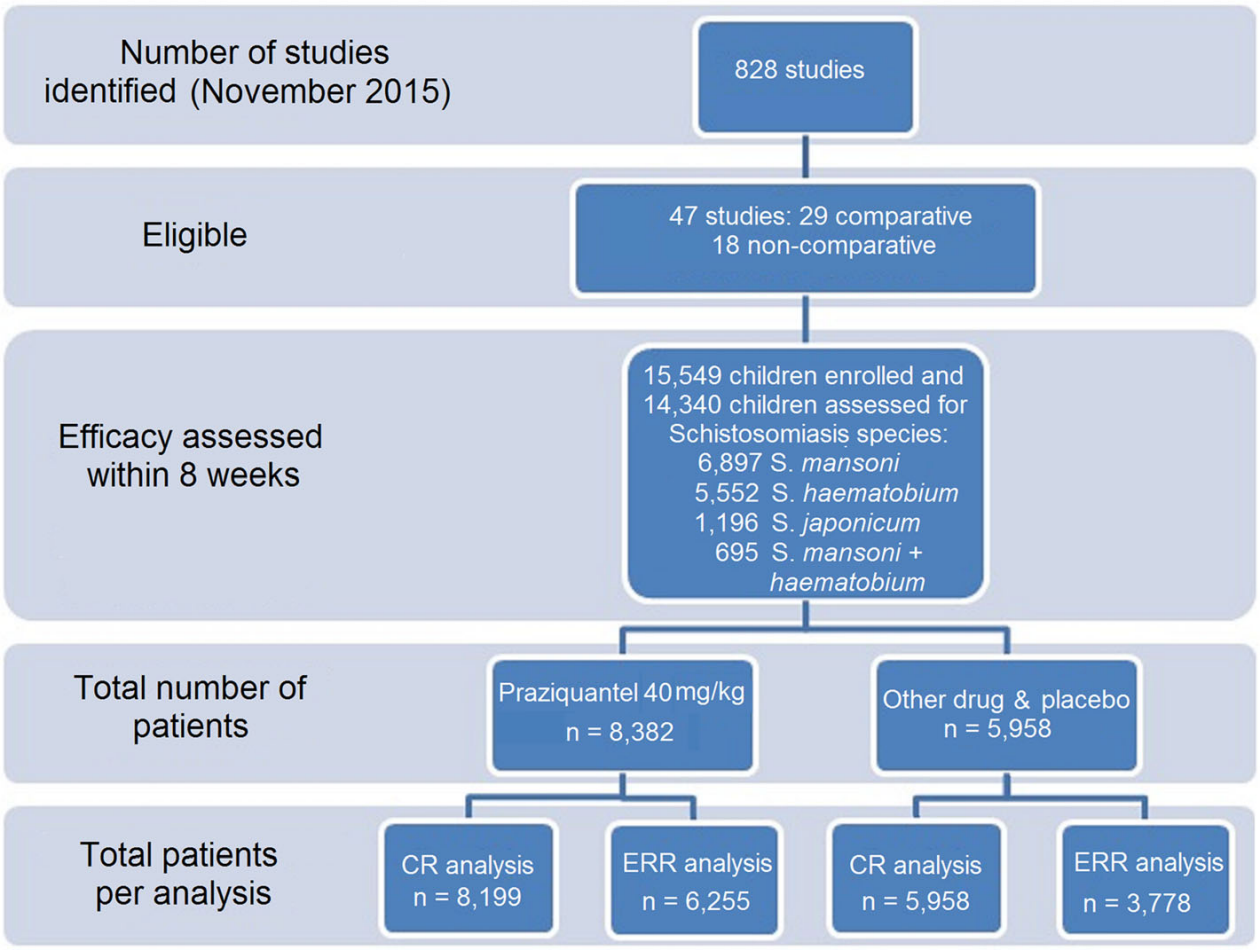
Flow chart of studies and patients

Treatment outcome was reported as CR in 14,157 children and as ERR (all calculations methods) in 10,033 patients overall, of whom 58% (*n* = 8,199) and 62% (*n* = 5,958), respectively, were treated with PZQ 40 mg/kg (Fig. 1). The breakdown by species and reported outcome was: both CR and ERR, 62% of the subjects; CR only, 33%; and ERR only, 5%. For *S. haematobium*, the outcome was reported as CR for 4,612 patients and 3,963 for ERR; for *S. mansoni* this was 6,897 and 5,138, respectively; for *S. mansoni/S. haematobium*, 695 and 111; and for *S. japonicum*, 1,196 and 403.

Of the 36 studies reporting outcomes as ERR, 31 used the ERRgm and 5 the ERRam (2 studies reported both) (Table 2). The ERRgm was calculated using a log-transformation applied to the entire per-protocol population for 24% of subjects, on uncured patients only in 16%, and not specified in 26%. No study reported ERRam on uncured subjects.

**Table 2:** Methods used in the studies to calculate egg reduction rate (ERR). Seven studies not presented in the table assessed S. *japonicum* or *S. haematobium*/*S. mansoni*

ERR calculation method at endpoint	No. of patients enrolled		No. of patients assessed		Number of studies					
					Total		*S. haematobium*		*S. mansoni*	
	*n*	%	*n*	%	*n*	%	*n*	%	*n*	%
ERRgm log-transformed	3,670	24	3,475	32	9	19	6	33	3	14
ERRam	881	6	715	7	5	11	2	11	3	14
ERRgm method unclear	3,980	26	3,790	23	12	26	4	22	6	27
ERRgm calculated on uncured	2,444	16	2,066	14	10	21	3	17	5	23
No ERR	4,574	29	4,294	30	11	23	3	17	5	23
Total	15,549	100	14,340	100	47	100	18	100	22	100

*Abbreviations: ERRam* egg reduction rate calculated using arithmetic mean, *ERRgm* egg reduction rate using geometric mean

Egg counts were reported using different diagnostic approaches (number of specimens and tests): from one to three days and one to three stools per day tested in simple or duplicate or triplicate slides. The most frequent approach for *S. mansoni* was one test on one stool sample collected on the same day and for *S. haematobium* was one test on two separate urine samples, collected on the same day. Subjects receiving PZQ 40 mg/kg for *S. mansoni* had a mean egg count before treatment of 376 eggs per gram of faeces (epg, range 1.2–2,662); the mean was higher in studies using arithmetic means (854, range 144–2,662 epg) than those using geometric means (276, range 1.2–1,065 epg); for *S. haematobium* 105 eggs per ml of urine (range 1–630), also higher with arithmetic than geometric means (120 and 108, respectively); and for *S. japonicum*, mean epg 297 (range 60–255; studies reporting only geometric means).

### Efficacy of PZQ 40 mg/kg

The calculated median age of the total 8,380 subjects treated with the PZQ 40 mg/kg was 9 years (range 1 month to 19 years) and clustered around 6 to 15 years of age. The median age of school-children varied widely across sites from 4 to 19 years, mostly depending on whether the study was school- or community-based.

**Fig. 2:**
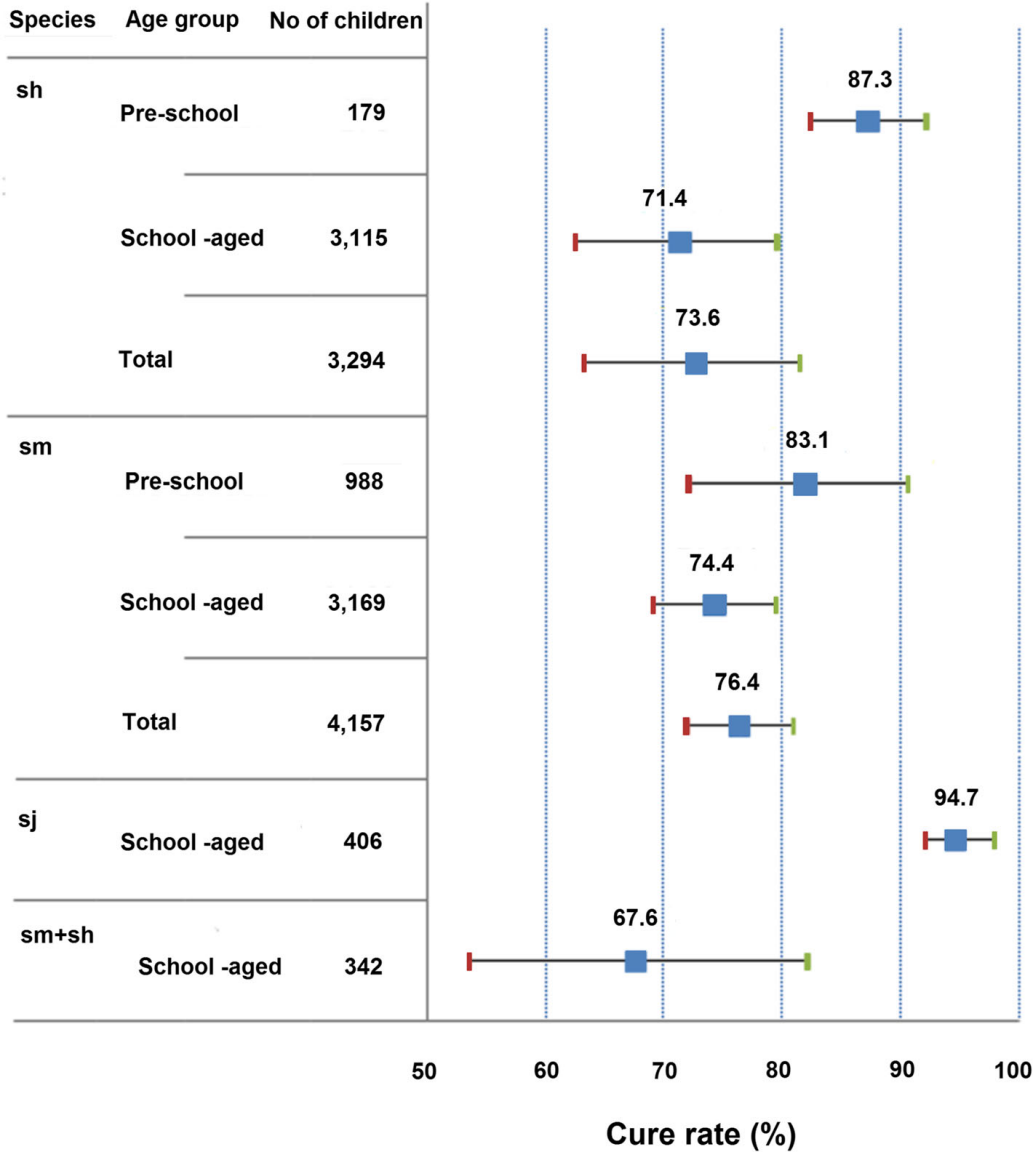
Cure rate (CR) by age group and *Schistosoma* species, PZQ 40 mg/kg. *Abbreviations*: sh, *S. haematobium*; sj, *S. japonicum*; sm, *S. mansoni*

The overall CR in children treated with PZQ 40 mg/kg (tablet and syrup) was 73.6% (95% CI: 64.1–82.3, 25 study groups, 3,294 children) for *S. haematobium* and 76.4% (95% CI: 71.5–81.0, 34 groups, 4,157 children) for *S. mansoni* (Fig. 2)*.* The overall reported ERRgm for *S. haematobium* was 90.9% (95% CI: 86.0–95.6, 21 groups, 2,586 children) but was 81.0% for ERR based on uncured patients and 99.5% for ERRgm log-transformed; for *S. mansoni* it was 84.3% (95% CI: 79.0–89.3, 22 groups, 3,233 children)*,* from 79.7% for ERR based on uncured patients to 92.0% for ERRgm log-transformed. For *S. japonicum* the CR was 94.7% (95% CI: 92.1–98.0, three groups, 406 children) and the ERRgm was 95.0% (95% CI: 90.1–99.9, one study, 203 children); in mixed *S. haematobium/S. mansoni* infections, the CR was 67.6% (95% CI: 53.6–82.4, three studies, 342 children) and the ERRgm 98.0% (95% CI: 90.9–99.9, one study, 54 children).

**Fig. 3:**
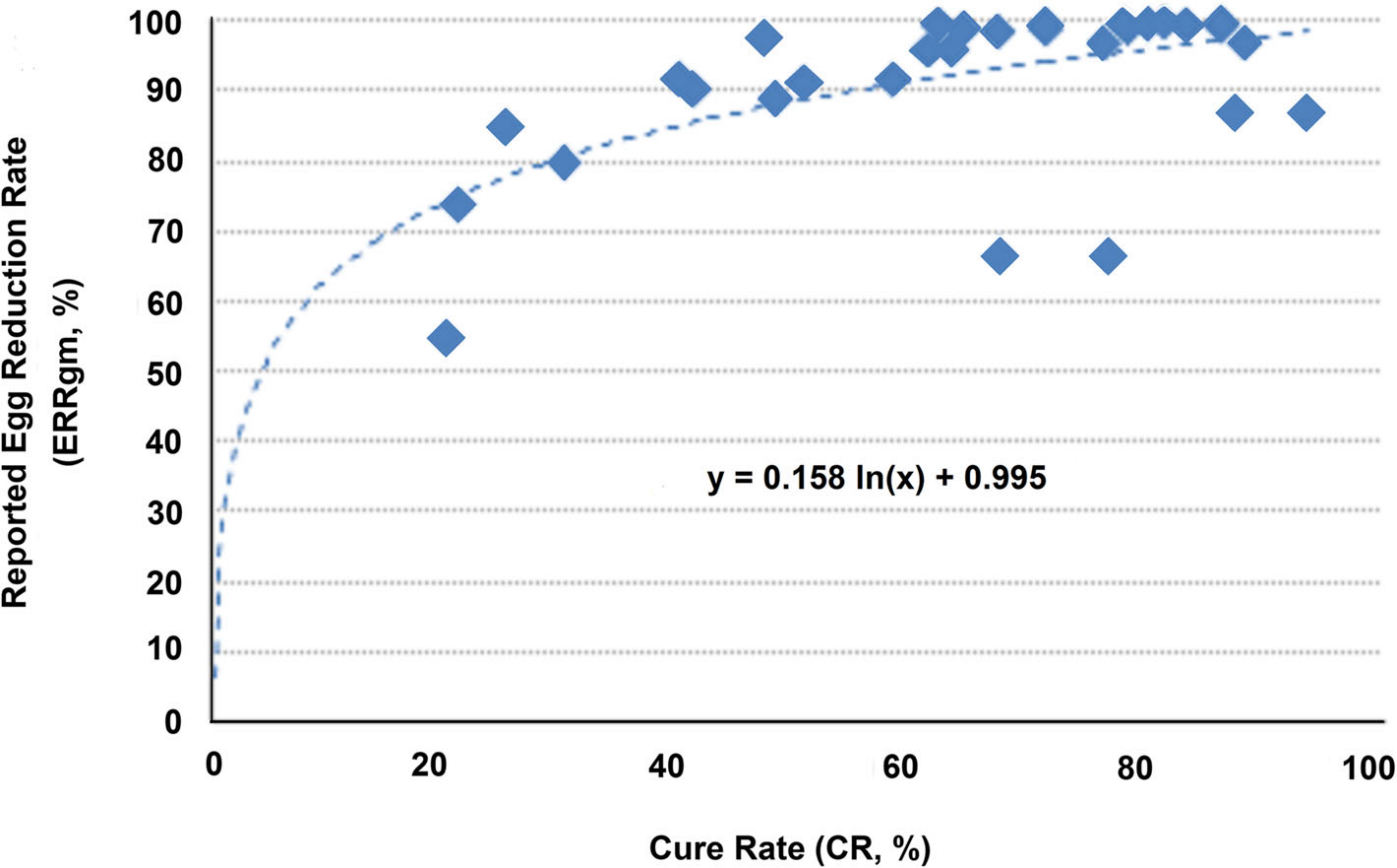
Correlation between cure rates (CR) and reported egg reduction rate using geometric mean with log-transformation (ERRgm)

Using the reported CR and ERRgm with log-transformation calculation from 34 study arms (Fig. 3), a logarithmic correlation was found (y = 0.158 ln(x) + 0.995, *R* ^2^ = 0.34) with which we extrapolated the corresponding predicted ERRgm (pERRgm) for all PZQ groups, whatever the original calculation method. As a result, in PZQ 40 m/kg groups, the overall pERRgm was 94.7% (95% CI: 92.7–96.4) for *S. haematobium*, 95.3% (95% CI: 94.2–96.2) for *S. mansoni*, 93.4% (95% CI: 89.9–96.2) for *S. mansoni*/*S. haematobium,* and 98.7% (95% CI: 98.3–99.2) for *S. japonicum* (Table 3).

**Table 3:** Efficacy, cure rate (CR) and predicted egg reduction rate using geometric mean (pERRgm) by *Schistosoma* species by age group and PZQ dose

Species	Age category	PZQ dose (mg/kg)	No. of study arms	No. of patients assessed	Reported CR			Reported ERR	Predicted ERR			Ratio ERR reported/predicted
					%	95% CI bootstrap		%	%	95% CI bootstrap		
						Lower	Upper			Lower	Upper	
sh	Pre-school	40	1	18	88.9	74.4	99.9	98.0	97.7	94.9	99.5	1.00
		syrup 40	1	161	85.7	80.3	91.1	69.4	97.1	96.1	98.1	1.40
		Total 40	2	179	87.3	85.7	88.9	83.7	97.4	97.1	97.7	1.16
	School-aged	30	2	84	82.7	71.0	94.3		96.5	94.1	98.6	
		40	23	3,159	72.4	63.6	81.2	90.9	94.4	92.4	96.3	1.04
		60	2	77	64.8	63.6	65.9		92.7	92.4	93.0	
		Total 40	25	3,338	73.6	64.8	81.7	90.9	94.7	92.7	96.4	1.04
sm	Pre-school	40	7	806	83.1	71.1	94.3	89.7	96.6	94.2	98.6	1.08
		syrup pzq40	2	182	78.0	75.0	80.9	77.9	95.6	95.0	96.2	1.23
		2*40	4	339	88.7	80.0	98.7	99.3	97.6	96.0	99.3	0.98
		Total 40	9	988	82.0	72.6	89.9	83.1	96.4	94.5	97.9	1.16
	School-aged	10	1	61	26.2	15.2	37.2		78.4	69.8	84.0	
		20	4	385	40.3	24.9	55.0	91.8	85.2	77.6	90.1	0.93
		30	1	197	63.0	56.3	69.7	96.1	92.3	90.5	93.9	0.96
		40	25	3,169	74.4	68.8	79.5	84.6	94.9	93.6	95.9	1.12
		60	5	667	78.6	67.8	90.0	85.1	95.7	93.4	97.9	1.13
		Total 40	34	4,157	76.4	71.5	81.0	84.3	95.3	94.2	96.2	1.13
sm + sh	School-aged	10	2	73	11.2	4.2	18.1		64.9	49.5	72.6	
		20	2	61	38.4	36.7	40.0		84.4	83.7	85.1	
		30	2	72	51.8	31.4	72.2		89.2	81.3	94.4	
		40	5	342	67.6	54.1	80.7	98.0	93.4	89.9	96.2	0.95
sj	School-aged	40	3	406	94.7	92.2	98.0	95.0	98.7	98.3	99.2	1.04
		60	2	200	97.5	97.0	98.0	95.4	99.1	99.0	99.2	1.04

*Abbreviations: ERR* egg reduction rate, *pERRgm* predicted egg reduction rate using geometric mean, *CR* cure rate, *CI* confidence interval, *sh S. haematobium*, *sj S. japonicum*, *sm S. mansoni*

The ratio between predicted and reported log-transformed ERRs averaged 1.1 (0.93–1.4). The sensitivity analysis restricted to the studies reporting log-transformed ERRgm showed that the pERRgm was on average 0.8% higher than the reported ERRgm (using logarithmic transformation). Similarly, the pERRgm was on average 24% higher than the reported ERRgm based on uncured patients, 12% higher than the reported ERRgm with unspecified method and 3% higher than the reported ERRam.

Based on reported ERRs (all methods), 45% (26/47) of the study arms treated with PZQ 40 mg/kg for any species would not meet the 90% ERR threshold, compared to 15% (7/47) after statistical correction (Additional file 3: Table S3).

### Efficacy of PZQ 40 mg/kg by species and age groups

#### *Schistosoma haematobium*

The CR was 87.3% (95% CI: 85.7–88.9, 2 groups) in preschool-aged children, and 71.4% (95% CI: 62.3–80.5, 22 groups) in school-aged children; the pERRgm was 97.1% (95% CI: 97.1–97.7) and 94.4% (95% CI: 92.4–96.3), respectively. The mixed-effect model did not detect any difference in CR or pERRgm between preschool- and school-aged children (*P* = 0.309, *P* = 0.490, respectively), syrup and tablet formulation (*P* = 0.925, *P* = 0.949), week of assessment (*P* = 0.287, *P* = 0.337), light *vs* more intense infection (*P* = 0.199, *P* = 0.086), one *vs* multiple samples (*P* = 0.723, *P* = 0.660).

#### *Schistosoma mansoni*

The CR was 82.0% (95% CI: 72.6–90.0, 9 groups) in preschool-aged children, and 74.4% (95% CI: 68.8–79.5, 25 groups) in school-aged children; the pERRgm was 96.4% (95% CI: 72.6–90.0) and 94.9% (95% CI: 93.6–95.9), respectively. The mixed-effect model did not detect any difference in CR or pERRgm between preschool- and school-aged children (*P* = 0.982, *P* = 0.895, respectively), syrup and tablet formulation (*P* = 0.956, *P* = 0.959), week of assessment (*P* = 0.791, *P* = 0.784), light *vs* more intense infection (*P* = 0.932, *P* = 0.837), one *vs* multiple samples (*P* = 0.741, *P* = 0.631).

#### *Schistosoma mansoni*/*S. haematobium*

The CR was 71.9% (95% CI: 61.8–87.7) in school-aged children under 10 years old in three studies, and 61.0% (95% CI: 45.5–76.6) in older school-aged children in two studies. The ERR was only recorded in one study for under 10’s and was 98.0% (95% CI: 94.6–100). The overall CR was 67.6% (95% CI: 54.1–80.7, 5 groups) and the corresponding pERRgm was 93.4% (95% CI: 89.9–96.2). Using multivariate analysis, no correlation was detected between age and CR (*P* = 0.625).

#### *Schistosoma japonicum*

The CR was 92.1% (95% CI: 86.8–97.4) in school-aged children under 10 years old in one study, and 96.0% (95% CI: 94.0–98.0) in older schoolchildren in two studies. The overall CR was 94.7% (95% CI: 92.2–98.0), the reported ERR was 95.0% (95% CI: 90.1–99.9) and the pERRgm was 98.7% (95% CI: 98.3–99.2).

### PZQ 40 mg/kg *vs* comparators

In studies comparing PZQ 40 mg/kg to other PZQ regimens for treating *S. haematobium*, and using meta-analysis regression (Additional file 4: Figure S1), no difference was detected *vs* PZQ 30 mg/kg (RR = 0.94, 95% CI: 0.87–1.01, *P* = 0.106) and split-dose PZQ 2*20 mg/kg (RR = 0.98, 95% CI: 0.94–1.04, *P* = 0.590); subjects treated with PZQ 40 mg/kg were at a significantly lower risk of failure than artesunate alone (RR = 0.54, 95% CI: 0.36–0.83, *P* = 0.005) or in combination (AS + SP, RR = 0.83, 95% CI: 0.79–0.88, *P* = 0.001; AS + MQ, RR = 0.69, 95% CI: 0.47–1.02, *P* = 0.066), mefloquine alone (RR = 0.24, 95% CI: 0.10–0.58, *P* = 0.001); metrifonate 10 mg/kg (RR = 0.15, 95% CI: 0.04–0.58, *P* = 0.005), albendazole (RR = 0.43, 95% CI: 0.30–0.61, *P* = 0.001). There was no difference between PZQ 40 mg/kg and PZQ 60 mg/kg, *P* = 0.750; ASMQ + PZQ, *P* = 0.906; PZQ + MQ, *P* = 0.499; albendazole + PZQ 40 mg/kg, *P* = 0.561. Only PZQ 40 mg/kg in combination with artesunate performed better than PZQ 40 mg/kg (RR = 1.17, 95% CI: 1.03–1.33, *P* = 0.019).

Similarly, patients treated with PZQ 40 mg/kg for *S. mansoni* (Additional file 5: Figure S2), were at a significantly lower risk of failure than lower doses of PZQ (10 mg/kg, RR = 0.35, 95% CI: 0.22–0.55; 20 mg/kg, RR = 0.56, 95% CI: 0.41–0.75; 30 mg/kg, RR = 0.81, 95% CI: 0.70–0.92); artesunate combined with SP (RR = 0.59, 95% CI: 0.46–0.75, *P* = 0.001) or artesunate with sulfalene (RR = 0.21, 95% CI: 0.12–0.34, *P* = 0.001), mirazid 300 mg/kg (RR = 0.15, 95% CI: 0.06–0.35, *P* = 0.001), albendazole (RR = 0.60, 95% CI: 0.43–0.84, *P* = 0.003), arachidonic acid (RR = 0.72, 95% CI: 0.55–0.96, *P* = 0.023). No difference was detected between PZQ 40 mg/kg formulated as tablet or syrup (RR = 1.01, 95% CI: 0.88–1.15, *P* = 0.884), PZQ 60 mg/kg (RR = 1.05, 95% CI: 0.98–1.12, *P* = 0.141), PZQ 2*40 mg/kg (RR = 1.03, 95% CI: 0.96–1.10, *P* = 0.401), PZQ 40 mg/kg + albendazole (RR = 1.01, 95% CI: 0.78–1.31, *P* = 0.921), PZQ + arachidonic acid (RR = 1.29, 95% CI: 0.88–1.88, *P* = 0.193) and oxamniquine at 15, 30, 40, 50 mg/kg (*P* = 0.254, *P* = 0.744, *P* = 0.080, *P* = 0.056, respectively).

No difference was detected in mixed *S. mansoni*/*S. haematobium* infection between PZQ 40 mg/kg and PZQ 10 mg/kg (RR = 0.15, 95% CI: 0.02–1.08, *P* = 0.060), 20 mg/kg (RR = 0.63, 95% CI: 0.35–1.15, *P* = 0.135), 30 mg/kg (RR = 0.87, 95% CI: 0.67–1.12, *P* = 0.278), split dose 2*20 mg/kg (RR = 1.07, 95% CI: 0.88–1.31, *P* = 0.494), and oltipraz 30 mg/kg (RR = 1.05, 95% CI: 0.92–1.19, *P* = 0.481).

No difference was detected in patients treated for *S. japonicum* between PZQ 40 mg/kg and PZQ 60 mg/kg (RR = 1.02, 95% CI: 0.97–1.07, *P* = 0.461) or PZQ 40 mg/kg + albendazole (RR = 0.99, 95% CI: 0.94–1.04, *P* = 0.767).

### Safety: adverse events (AEs)

Overall, 22 studies recorded AEs in 6,713 children. One additional tolerability study was included [68] to the 21 studies considered for the efficacy analysis. The proportion of children treated with PZQ 40 mg/kg was 67% (*n* = 4,480), 22% of whom were preschool-aged (*n* = 980). The syrup formulation was tested on half of the pre-school aged children. Out of the three studies reporting AEs, one compared PZQ 40 mg/kg tablet and syrup formulation [24]. The two other studies were not comparative: one tested PZQ 40 mg/kg tablet [22] and the other one the PZQ 40 mg/kg liquid syrup formulation [23]. Five thousand, nine hundred and twenty-six AEs were reported within 48 h of treatment in PZQ 40 mg/kg recipients. Most of the signs and symptoms recorded were gastro-intestinal, neurological and dermatological.

**Fig. 4:**
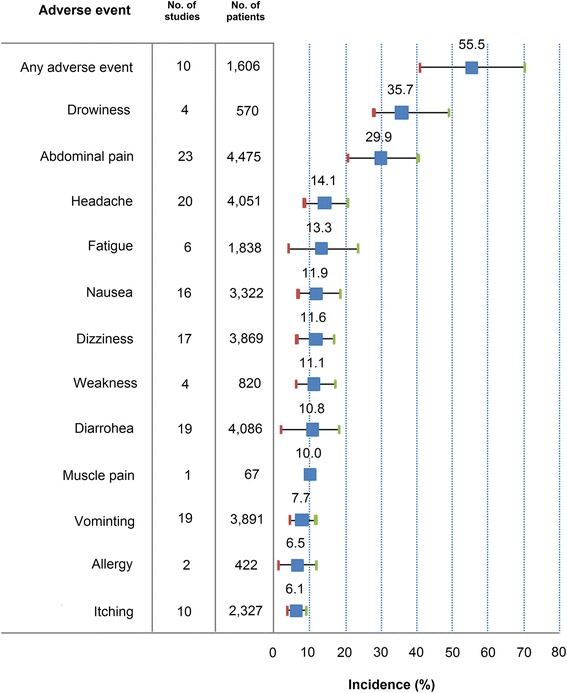
Adverse event incidence, preschool and school children, PZQ 40 mg/kg

On average 55.5% (95% CI: 39.4–78.2) of the 1,606 patients treated in 10 studies with PZQ 40 mg/kg experienced at least one of the AEs listed (Fig. 4). Signs and symptoms were not sought systematically in all studies. The incidence of AEs ranged from 6.1% for itching/rash (studied in 10 studies) to 35.5% for drowsiness (studied in 3 studies). In preschool-aged children in Uganda [18], no significant difference was detected between PZQ 40 mg/kg tablet (*n* = 254) and syrup (*n* = 249) in 15 different signs or symptoms screened.

One study analyzed age trends in schoolchildren aged between 6 and 19 years old [46]. Authors found more AEs in older subjects (*P* = 0.008) and differences between sites.

### Summary of efficacy and safety finding in trials in preschool-aged children: a narrative review

Five studies [16–20] enrolled the totality of the 1,694 preschool-aged children, of whom 89% (*n* = 1,506) were assessed for efficacy, 988 for *S. mansoni* and 179 for *S. haematobium* treated with PZQ 40 mg/kg (90% with tablet formulation).

In Navaratnam et al. [24], 203 preschool-aged children aged 1.5 to 5 years were randomly assigned to PZQ 40 mg/kg (crushed) tablet or syrup formulation. The pre-treatment arithmetic mean egg count was 317.6 in the tablet group and 292.1 in the syrup group. No difference was detected between the syrup and tablet formulations for CR (80.9 *vs* 81.7%) and ERRam (86.1 *vs* 89.0%). There was a significant correlation between pre-treatment infection intensity and efficacy when assessed as CR: 88.6% in light, 74.5% in moderate and 67.4% in heavy infection. Non-compliance to PZQ syrup was 11.1% (95% CI: 8.6–14.0) and that of PZQ tablet was 14.7% (95% CI: 12.0–18.0) with no significant difference detected (OR = 1.33, 95% CI: 0.93–1.92, *P* =0.110). Infants 12 months of age were 20.3 times more likely (*P* = 0.001) to react negatively to the syrup formulation than 5 year olds. Amelioration of vomiting (OR = 1.65, *P* = 0.006) and peri-rectal bleeding (OR = 1.40, *P* = 0.007) was significantly higher with crushed PZQ tablet. The most common AEs in both treatment arms were dizziness, drowsiness and fatigue. This was the only study to report dizziness in preschool-aged children, and the incidence seemed to be higher than what reported in 13 studies in school-aged children (Table 4).

**Table 4:** Adverse event incidence by age group, PZQ 40 mg/kg

Adverse event	Pre-school aged					School-aged				
	No. of study arms	No. of subjects	Incidence (95% CI)			No. of study arms	No. of subjects	Incidence (95% CI)		
			%	Lower bound	Upper bound			%	Lower bound	Upper bound
Any adverse event (at least one)	1	243	34.2	28.2	40.2	8	1,342	54.2	37.0	72.2
Drowsiness	2	503	29.1	28.0	30.1	2	285	42.4	35.8	49.0
Dizziness	3	737	22.4	1.3	33.9	13	3,132	9.3	5.4	14.1
Fatigue	3	737	13.3	0.9	20.9	3	781	13.2	0.4	28.9
Itching/rash	2	503	8.6	8.0	9.1	8	1,504	5.5	2.3	9.3
Nausea	3	737	7.8	2.1	12.6	12	3,058	12.9	6.4	20.2
Abdominal pain	3	737	7.2	3.0	9.4	19	3,495	33.4	22.6	43.9
Vomiting	3	737	6.8	1.7	9.6	15	2,911	7.9	3.8	13.2
Headache	3	737	4.4	0.4	6.7	17	3,314	16.6	9.8	24.0
Diarrhoea	3	737	4.3	2.6	6.3	16	3,106	12.1	6.5	19.3

*Abbreviations: CI* confidence interval

In Sousa-Figueiredo et al. [26], 305 patients aged from 5 months to 7 years were treated for *S. mansoni*. The overall pre-treatment egg count was 144.1 or 5.99 eggs using the arithmetic and geometric mean respectively. Children under 4 years-old had a lower CR (40.8%) and ERRam (75.4%), than 4 to 7 years-old (60.8 and 82.9%, respectively). Children with history of previous treatment had a lower CR (41.7%) and ERRam (72.8%) than treatment-naive children (77.6 and 92.1%, respectively). PZQ tablets proved to be safe, with only mild, transient reported adverse events. At the6-month and 12-month follow-up times, the number of cases of dizziness, sleepiness, fatigue, cramps, nausea, sweating and night fevers post-treatment decreased significantly compared to baseline. No symptom became more prevalent with time.

In Nalugwa et al. [25], Ugandan preschool-aged children aged from 12 to 60 months were randomized to receive PZQ 40 mg/kg or 2*40 mg/kg tablets (2 weeks apart). By week 4, no difference was detected between treatment for CR (83.2 and 85.5%, *P* = 0.390, respectively) or ERRgm (98.9 and 99.3%, respectively). Efficacy was lower in children with higher infection intensity and higher in younger children.

Two studies treated preschool-aged children for *S. mansoni* and *S. haematobium* with PZQ 40 mg/kg tablet (Côte d’Ivoire, *n* = 53 [16]) and syrup (Niger, up to 67 months, *n* = 249 [17]). In Côte d’Ivoire the reported efficacy was high against both *S. mansoni* (CR, 88.6%; ERR, 96.7%) and S. *haematobium* (CR, 88.9%; ERR, 98.0%); in Niger, the reported efficacy was comparatively lower for both *S. haematobium* (CR, 85.7%; ERR, 69.4%) and *S. mansoni* (CR, 75.0%; ERR, 66.7%). Both trials reported only mild AEs (only one case of face and body inflammation that was graded as a moderately severe, [16]) and no serious AE.

## Discussion

This analysis is based on a systematic review of comparative and non-comparative studies of PZQ for treating intestinal and urinary schistosomiasis, and concentrates on the efficacy and safety of the WHO-recommended standard dose of 40 mg/kg in preschool- and school-aged children. The main question that we attempted to answer was whether efficacy varied with the subject’s age, and more specifically if PZQ at 40 mg/kg is as effective in preschool-aged children as in older children, so that it can be used more broadly in this younger age category. These analyses do not indicate any age-effect on efficacy, whether expressed as CR or ERRgm, or a dose-effect between 40 and 60 mg/kg, for any *Schistosoma* species. However, one should bear in mind the limitations of this aggregate-data meta-analysis.

First, only five studies compared efficacy explicitly between age-groups [25, 26, 38, 52, 54]. Secondly, age trends could not be calculated reliably because age categories as reported in the papers are often broad and inconsistent across studies, so that the calculated median age may not reflect the range of responses within each individual group. Following the WHO age classification for PC, the preschool-aged children age-group should include 1–4-year-olds and the school-aged group 5–14-year-olds. Thirdly, preschool-aged children are under-represented: 11% of all subjects treated with PZQ 40 mg/kg. Lastly, and importantly, methodology was insufficiently standardized: studies differed in terms of diagnostic approach used (number of samples and smears), outcome measures (CR, ERR using geometric or arithmetic mean), duration of follow-up, and safety reporting. ERR was predominantly calculated using geometric means, which are less apt to identify outliers (patients responding less well) and tend to show higher efficacy, compared to arithmetic means or individual-patient response distributions [3, 69]. It is therefore difficult to apply the WHO-recommended threshold of 90% ERRam [3] to the results of these studies and meta-analysis. Moreover, when the geometric mean egg count was used, some studies calculated the ERR using the geometric mean as the difference between egg counts on all the patients, pre-treatment, and only the positive subjects at the time of the endpoint assessment. This method is inappropriate and biases the result because the denominator changes from the baseline to the endpoint time, overestimates the egg counts post-treatment and underestimates efficacy (in our calculations here by ~ 24%).

To mitigate these shortcomings, we recalculated the ERRgm and applied multivariate analyses. The predicted ERRgm (pERRgm) provided a good fit with the reported ERRgm using log-transformation; only three data points (the lowest reported ERRgm) out of 34 fell well below the trend line, which demonstrates a good regression fit. Of these, two are from the same study (*S. mansoni* infection, moderate intensity, Kenya [62]). There might be multiple reasons for this. One is mathematics (the geometric mean wipes out extreme values, so the contribution of individual outliers in the response distribution to the overall response is minimised). Another one is genetic heterogeneity between schistosome populations from different geographical regions, which means that otherwise generalizable trends might not apply to some specific local situations. A third one is the background prevalence of infection (which could not be estimated here, but appears to play an important role [10]). Predicting ERRgm from CR has therefore its limitations, but it allowed including a broader range of studies and a larger number of subjects that would have otherwise been possible, making it possible to expand the utility and comparability of older studies; it is expected that this will not be required with newer studies adopting the WHO recommendation to express results as ERRam [3].

With respect to the WHO-recommended threshold of 90% efficacy by ERRam, and with all the above-mentioned provisos, using our statistical correction, we found that 85% of the study arms treated with PZQ 40 mg/kg for any species would meet this criterion.

The multivariate analyses with random effect on the study site to account for differences between studies using PZQ 40 mg/kg in S. *mansoni* or S. *haematobium* found no relationship between efficacy (whether measured as CR or ERR), and age (preschool *vs* school children aged), duration of follow-up (one *vs* two months), formulation (syrup *vs* tablet), diagnostic approach (single *vs* multiple smears), and infection intensity (light *vs* others). It is however worth noting that the effects of risk factors on efficacy varied across individual studies. Sousa-Figueiredo et al. (Uganda, *S. mansoni*) [26] found that the risk of not clearing the infection was significantly higher in children less than four years-old than four to seven year-old by a factor of 3.5, and in children with heavy infections by a factor of circa two. Thiong’o et al. [62] (Kenya, 5–9, 10–14, 15+ years) found a significant trend in four study sites for efficacy measured as CR to decrease with age: ranging 59–92, 72–79, 80–93, 52–78%, and ERR ranging 99–100, 99–100, 93–98, 75–91); of note, older children were more heavily infected and had higher ERRgm than younger children with lower infection intensity before treatment. Gryseels et al. [54] in Burundi found opposite trends in CRs after treatment with doses of 20, 30 and 40 mg/kg in subjects aged < 20 (from ~ 52 to 78%) and 20+ years (from 80 to ~ 94%). A recent study conducted on 508 Zimbabwean children treated with PZQ 40 mg/kg for *S. haematobium* not included in this review [70] found no difference in CR and ERR between children aged one to five (both 100%) and six to 10 years (94% and 97.9%, respectively). A previous systematic review and meta-analysis [10] which compared CR in similar numbers of participants (1,026 preschool-aged and 12,906 school-aged children), also did not detect significant differences in the combined CRs for *S. mansoni* and *S. haematobium* (73.6 *vs* 71.1%, respectively), but rather found a correlation between lower CR and higher background infection prevalence, especially for younger children. Here, we confirmed and extended these findings by including also ERRgm (see above), though both CR and ERRgm are probably inaccurate for the reasons listed above. These disparate findings likely reflect a range of situations in terms of infection prevalence and intensity in younger and older children which also depends on the deployment and coverage of preventive chemotherapy cycles in the population.

These findings must be considered in the light of the current imperfect test-of-cure; counting eggs in excreta (using the available low-sensitivity methods) cannot distinguish between definitive (adult worm killing) and temporary effects (suspended egg excretion). These methods, while useful in quantifying effects of PC programmes at the population level, are inaccurate when it comes to assess the real drug efficacy and detect early signals of reduced efficacy and potential resistance.

The other question was whether higher levels of efficacy could be achieved using 60 mg/kg instead of 40 mg/kg (and in which age-group). No evidence of difference emerged from this meta-analysis in children or previous meta-analyses in overall population [12–14]. However, a definitive conclusion cannot be reached on this question, both because the numbers are relatively small (*S. mansoni*: PZQ 40 mg/kg, *n* = 492 *vs* PZQ 60 mg/kg, *n* = 473; *S. haematobium*: PZQ 40 mg/kg, *n* = 74 *vs* PZQ 60 mg/kg, *n* = 77), and because ERRs do not capture the distribution of individual responses, and particularly outliers [69]. We also know that better efficacy can be achieved with longer treatment and higher doses - conditions that are however not feasible in routine PC.

Compared to our previous systematic review and meta-analysis, this was focused on the efficacy and safety of PZQ in children; here, the calculated median age was 10 years old, and the range across all studies was one to 19 years, for a total of 14,340 subjects with an efficacy outcome. The previous analysis (*n* = 19,499) had all treatments and age-groups (age range 1–75 years old). In addition, here we explored different approaches for ERR calculations methods and applied a statistical correction to standardize ERR outcomes.

Praziquantel was well tolerated in all age groups, though approximately half of the subjects experienced an adverse event. It must be however noted that safety was variably and inconsistently assessed; few AEs were assessed systematically across most studies. The frequency of certain AEs in each age-group may simply reflect the fact that these were recorded in certain studies, and not others. Also, it is very difficult to detect true signal from background noise: the absence of pre-exposure information on presence and severity of signs/symptom, prevents assessing whether the events reported were related or not to treatment. It is all-important that clinical researchers be reminded of the critical relevance of collecting safety information in the controlled environment of clinical trials, even for drug which are widely regarded as well-tolerated: this is especially true for drugs, like praziquantel, that are used predominantly in children, who might not complain and cannot verbalize discomfort, with the risk of risks being underrated events being underreported [71]. A simple way to doing that is to collect and grade signs and symptoms at enrolment to allow comparison between pre- and post-drug exposure, to identify treatment-emergent signs and symptoms. Child-friendly questionnaires including drawings should be considered, especially in young children.

## Conclusions

In summary, PZQ at the standard dose of 40 mg/kg was generally effective at reducing intensity infection of all *Schistosoma* species at all ages of those studied, but efficacy varied across studies and age-ranges. This conclusion should be tempered to reflect the above-mentioned limitations and will require further studies and pooling of data for an individual patient data meta-analysis. It is indeed possible that exposure to infection and infection intensity increase with age in the absence of effective PC. This confounds analysis and makes it difficult to conclude reliably as to whether the same PZQ dose should be applied in preschool-aged children. It is also worth mentioning that these conclusions have practical implications for the solid oral form of praziquantel, as other formulations are not recommended for use by the WHO. This report also highlights the need for consistent methodologies to be used in designing clinical trials for schistosomiasis to assess and report on efficacy and safety.

## Additional files

Additional file 1: Table S1. PRISMA checklist. (DOC 70 kb)
Additional file 2: Table S2. Study design characteristics. (DOCX 20 kb)
Additional file 3: Table S3. Reported efficacy and predicted egg reduction rate by age group and site, PZQ 40 mg/kg. (DOCX 32 kb)
Additional file 4: Figure S1. Forest plot for PZQ 40 mg/kg and comparator treatments for *S. haematobium*, cure rate (CR) and risk ratio (RR). *Abbreviations*: pzq40, praziquantel 40 mg/kg; pzq20, praziquantel 20 mg/kg; pzq30, praziquantel 30 mg/kg; pzq60, praziquantel 60 mg/kg; RR, risk ratio; comp, comparator group; AS, artesunate; MQ, mefloquine; ASMQ, artesunate + mefloquine; ASSP, artesunate + sulfadoxine-pyrimethamine; met10, metrifonate 10 mg/kg. (TIF 7292 kb)
Additional file 5: Figure S2. Forest plot for PZQ 40 mg/kg and comparator treatments for *S. mansoni*, cure rate (CR) and risk ratio (RR)*.* I2 is calculated for pooled subgroups as = 100%*(Q - df)/Q, where Q is Cochran’s heterogeneity statistic and df the degrees of freedom. *Abbreviations*: pzq40, praziquantel 40 mg/kg; pzq10, praziquantel 20 mg/kg; pzq20, praziquantel 20 mg/kg; pzq30, praziquantel 30 mg/kg; pzq60, praziquantel 60 mg/kg; pzq syrup, praziquantel syrup 40 mg/kg; ARA arachidonic acid; ox, oxamniquine; RR, risk ratio; comp, comparator group; I2, Higgins’ I squared. (TIF 8364 kb)

## Abbreviations

AE: Adverse event
AQ: Amodiaquine
ARA: Arachidonic acid
AS: Artesunate
CI: Confidence interval
CR: Cure rate
epg: Eggs per gram
ERR: Egg reduction rate
ERRam: Egg reduction rate using arithmetic mean
ERRgm: Egg reduction rate using geometric mean
OX: Oxamniquine
PC: Preventive chemotherapy
pERRgm: Predicted egg reduction rate using geometrical mean
PRISMA: Preferred reporting items for systematic reviews and meta-analyses
PZQ: Praziquantel
RR: Risk ratio
sh: *S. haematobium*
sj: *S. japonicum*
sm: *S. mansoni*
SP: Sulfadoxine-pyrimethamine
WHO: World Health Organization
