# Supplementary material for: Efficacy and safety of praziquantel 40 mg/kg in preschool-aged and school-aged children: a meta-analysis
Source: Parasit Vectors. 2017 Jan 26;10:47. doi: 10.1186/s13071-016-1958-7 (PMC5270314; doi:10.1186/s13071-016-1958-7)
Supplement: Additional file 2: Table S2. — Study design characteristics. (DOCX 20 kb) [file 13071_2016_1958_MOESM2_ESM.docx]

Additional file 2, Table S2: Study design characteristics

| Species | Publication | Country | Study design | | | | |
| --- | --- | --- | --- | --- | --- | --- | --- |
| Study reference | (name, year) | | Controlled | | Blinding | Details | |
| S. *haematobium* | |  |  | |  |  | |
| [27] | Borrmann 2001 | Gabon | cs | |  | community based, computer-generated randomization, other risks of randomisation bias unclear | |
| [28] | Davis et al. 1979 | Zambia | cs | | 2 | school based, Allocation concealment: double blind, low risks of randomisation bias | |
| [29] | de Clercq et al. 2002 | Senegal | cs | |  | school based, exhaustive, selective treatment study, unclear risks of randomisation bias | |
| [20] | Inyang-Etoh et al. 2009 | Nigeria | cs | |  | community based, exhaustive, placebo controlled, randomisation unclear | |
| [30] | Keiser et al. 2010 | Ivory coast | cs | |  | school based, exploratory open-label trial, computer-generated randomization code, unclear risks of other randomisation bias | |
| [31] | Keiser et al. 2014 | Ivory coast | cs | |  | school based, exhaustive, computer- generated randomization, unclear other risks of randomisation bias | |
| [32] | Latham et la. 1990 | Kenya | cs | |  | school-based, male only, unclear risks of randomisation bias | |
| [33] | McMahon and Kolstrup 1979 | Tanzania | cs | |  | unclear risks of randomisation bias | |
| [34] | Midzi et al. 2008 | Zimbabwe | sts | | na | school based, exhaustive, selective treatment study | |
| [35] | N'goran et al. 2003 | Ivory coast | sts | | na | School based, exhaustive, selective treatment study | |
| [36] | Oyideran et al. 1981 | Nigeria | cs | |  | placebo controlled, unclear risks of other randomisation bias | |
| [37] | Ojurongbe et al. 2014 | Nigeria | sts | |  | school based, selective treatment study, unclear risk of randomisation bias | |
| [38] | Ouldabdallahi et al. 2013 | Mauritania | cs | |  | community based, unclear risks of randomisation bias | |
| [39] | Senghor et al. 2015 | Senegal | sts | | na | community based, selective treatment study, unclear risk of randomisation bias | |
| [40] | Sissoko et al. 2009 | Mali | cs | | 1 | School based, block randomization, single blind, high quality | |
| [41] | Stete et al. 2012 | Ivory Coast | sts | |  | school based, selective treatment study, unclear risk of randomisation bias | |
| [42] | Tchuente et al. 2004 | Cameroon | sts | | na | school based, exhaustive, selective treatment study | |
| [43] | Wilkins and Moore 1987 | Gambia | sts | | na | computer-generated, unclear risks of randomisation bias and attrition bias incomplete | |
| S. *japonicum* |  |  |  | |  |  | |
| [44] | Belizario et al. 2008 | Philippines | cs | | 2 | school based, randomized, double blind | |
| [45] | Olliaro et al. 2011 | Philippines | cs | | 2 | double-blind trial, computerised block size of 4, low risk of bias | |
| S. *haematobium*, S. *japonicum*, S. *mansoni* | | | | | | |  |
| [46] | Olds et al. 1998 | Kenya, Phillipines, China | | cs | 2 | school based, double blind placebo controlled, multisite, randomised block design of size 80, low risk of bias | |
| S. *mansoni* |  |  | |  |  |  | |
| [47] | Barakat and El Morshedy 2011 | Egypt | | sts |  | school based, exhaustive, unclear risks of randomisation bias | |
| [48] | Barakat et al. 2015 | Egypt | | cs |  | school based, exhaustive, unclear risks of randomisation bias | |
| [49] | Berhe et al. 1999 | Ethiopia | | sts | na | school based, exhaustive, selective treatment study | |
| [50] | Botros et al. 2005 | Egypt | | cs |  | community based, stratified randomisation, unclear risks of randomisation bias | |
| [51] | Degu et al. 2002 | Ethiopia | | sts | na | school based, exhaustive, selective treatment study | |
| [52] | Erko et al. 2012 | Ethiopia | | sts |  | school based, exhaustive, unclear risks of randomisation bias | |
| [53] | Friis and Byskov 1989 | Botswana | | sts | na | school based, not exhaustive, selective treatment study, stratified randomisation | |
| [54] | Gryseels et al. 1987 | Burundi | | sts | na | community based, exhaustive, selective treatment study | |
| [55] | Guisse et al. 1997 | Senegal | | cs |  | community based, unclear risks of randomisation bias and attrition bias incomplete | |
| [56] | Massoud et al. 1984 | Egypt | | cs |  | school based, exhaustive, unclear risks of randomisation bias | |
| [57] | Metwally et al. 1995 | Egypt | | cs |  | school based, exhaustive, stratified randomisation, unclear risks of randomisation bias | |
| [58] | Mohamed et al. 2009 | Sudan | | cs |  | school based, open label, unclear risks of randomisation bias | |
| [25] | Nalugwa et al. 2015 | Uganda | | cs |  | community based, unclear risks of randomisation bias | |
| [24] | Navaratnam et al. 2012 | Uganda | | cs |  | community based, exhaustive, every second child assigned to the same treatment arm, other risks of randomisation bias unclear | |
| [59] | Obonyo et al. 2010 | Kenya | | cs | 1 | school based, open-label randomised trial, computer-generated block, single blind, unclear other risks of bias | |
| [38] | Ouldabdallahi et al. 2013 | Tanzania | | cs | 2 | double-blind trial, computerised block size of 4, sealed and numbered envelopes | |
| [60] | Selim et al. 2014 | Egypt | | cs |  | school based, unclear risks of randomisation bias | |
| [61] | Simonsen et al. 1990 | Ethiopia | | sts | na | school based, exhaustive, selective treatment study | |
| [26] | Sousa-Figueiredo et al. 2012 | Uganda | | sts | na | community based, exhaustive, selective treatment study | |
| [67] | Teesdale et al. 1984 | Malawi | | sts |  | school based, stratified randomisation, unclear risks of randomisation bias | |
| [64] | Thiongo'o et al. 2002 | Kenya | | cs |  | school based, exhaustive, unclear risk of randomisation bias and attrition bias incomplete | |
| [68] | Utzinger et al. 2000 tmih | Ivory coast | | sts | na | school based, selective treatment study, unclear risk of randomisation bias | |
| S. *mansoni* + S. *haematobium* | | | | | | |  |
| [22] | Coulibaly et al. 2012 | Ivory Coast | | sts |  | community based, exhaustive, selective treatment study | |
| [69] | El Tayeb et al. 1988 | Sudan | | cs |  | school based, exhaustive, randomisation on the toss of a coin, unclear other risks of randomisation bias | |
| [23] | Garba et al. 2013 | Niger | | sts |  | community based, exhaustive, selective treatment study | |
| [70] | Kardaman et al. 1985 | Sudan | | sts | na | school based, exhaustive, unclear risks of randomisation bias | |
| [71] | Taylor et al. 1988 | Zimbabwe | | cs | 1 | school based, exhaustive, single blind, unclear risk of other bias | |

Legend: sts, selective treatment study; cs, comparative study; na, non applicable.
