# Supplementary material for: Efficacy and safety of praziquantel 40 mg/kg in preschool-aged and school-aged children: a meta-analysis
Source: Parasit Vectors. 2017 Jan 26;10:47. doi: 10.1186/s13071-016-1958-7 (PMC5270314; doi:10.1186/s13071-016-1958-7)
Supplement: Additional file 3: Table S3. — Reported efficacy and predicted egg reduction rate by age group and site, PZQ 40 mg/kg. (DOCX 32 kb) [file 13071_2016_1958_MOESM3_ESM.docx]

Additional Table S3: Reported efficacy and predicted egg reduction rate by age group and site, PZQ 40 mg/kg

| Name, Year | Species | N assessed | Age group | Treatment | CR | Reported ERR | | | Predicted ERR (pERRgm) | | |
| --- | --- | --- | --- | --- | --- | --- | --- | --- | --- | --- | --- |
|  |  |  |  |  | % | % | > 90% | calculation method | % | reported/predicted | > 90% |
| Borrmann et al. 2001 | sh | 89 | school-aged | 40 mg/kg | 73.0% | (negative, na) | no | ERRgm uncured | 94.6% |  | yes |
| Coulibaly et al. 2012 | sh | 18 | preschool | 40 mg/kg | 88.9% | 98.0% | yes | ERRgm unclear | 97.7% | 1.00 | yes |
| Davis et al. 1979 | sh | 53 | school-aged | 40 mg/kg | 98.1% |  |  | na | 99.2% |  | yes |
| Davis et al. 1979 | sh | 45 | school-aged | 40 mg/kg | 100.0% |  |  | na | 99.5% |  | yes |
| de Clercq et al. 2002 | sh | 88 | school-aged | 40 mg/kg | 30.0% | 80.0% | no | ERRgm log-transformed | 80.6% | 1.01 | no |
| de Clercq et al. 2002 | sh | 45 | school-aged | 40 mg/kg | 76.0% | 97.0% | yes | ERRgm log-transformed | 95.2% | 0.98 | yes |
| Garba et al. 2013 | sh | 161 | preschool | syrup pzq40 | 85.7% | 69.4% | no | ERRgm uncured | 97.1% | 1.40 | yes |
| Inyang-Etoh et al. 2009 | sh | 42 | school-aged | 40 mg/kg | 73.0% | 79.3% | no | ERRgm unclear | 94.6% | 1.19 | yes |
| Keiser et al. 2010 | sh | 26 | school-aged | 40 mg/kg | 88.0% | 97.0% | yes | ERRgm log-transformed | 97.5% | 1.01 | yes |
| Keiser et al. 2014 | sh | 21 | school-aged | 40 mg/kg | 33.0% | 94.0% | yes | ERRgm unclear | 82.1% | 0.87 | no |
| Latham et al. 1990 | sh | 16 | school-aged | 40 mg/kg | 81.0% | 99.9% | yes | am | 96.2% | 0.96 | yes |
| McMahon and Kolstrup 1979 | sh | 35 | school-aged | 40 mg/kg | 83.0% | 99.6% | yes | ERRgm log-transformed | 96.6% | 0.97 | yes |
| McMahon and Kolstrup 1979 | sh | 30 | school-aged | 40 mg/kg | 86.0% | 99.8% | yes | ERRgm log-transformed | 97.2% | 0.97 | yes |
| Midzi et al. 2008 | sh | 624 | school-aged | 40 mg/kg | 88.5% |  |  | na | 97.6% |  | yes |
| N'goran et al. 2003 | sh | 354 | school-aged | 40 mg/kg | 93.0% | 73.6% | no | ERRgm uncured | 98.4% | 1.34 | yes |
| Ojurongbe et al. 2014 | sh | 245 | school-aged | 40 mg/kg | 49.4% | 57.1% | no | ERRgm unclear | 88.4% | 1.55 | no |
| Olds et al. 1998 | sh | 95 | school-aged | 40 mg/kg | 64.9% |  |  | na | 92.7% |  | yes |
| Ouldabdallahi et al. 2013 | sh | 26 | school-aged | 40 mg/kg | 61.5% |  |  | na | 91.9% |  | yes |
| Ouldabdallahi et al. 2013 | sh | 48 | school-aged | 40 mg/kg | 70.8% |  |  | na | 94.1% |  | yes |
| Oyidiran et al. 1981 | sh | 21 | school-aged | 40 mg/kg |  | 97.7% | yes | ERRgm log-transformed | 97.7% | 1.00 | yes |
| Oyidiran et al. 1981 | sh | 19 | school-aged | 40 mg/kg |  | 98.7% | yes | ERRgm log-transformed | 98.7% | 1.00 | yes |
| Senghor et al. 2015 | sh | 237 | school-aged | 40 mg/kg | 93.7% | 83.7% | no | ERRgm uncured | 98.5% | 1.18 | yes |
| Sissoko et al. 2009 | sh | 389 | school-aged | 40 mg/kg | 97.7% | 95.6% | yes | ERRgm unclear | 99.2% | 1.04 | yes |
| Stete et al. 2012 | sh | 90 | school-aged | 40 mg/kg | 92.9% | 99.0% | yes | am | 98.4% | 0.99 | yes |
| Tchuente et al. 2004 | sh | 246 | school-aged | 40 mg/kg | 39.8% | 91.9% | yes | ERRgm log-transformed | 85.0% | 0.93 | no |
| Tchuente et al. 2004 | sh | 134 | school-aged | 40 mg/kg | 41.0% | 90.4% | yes | ERRgm log-transformed | 85.5% | 0.95 | no |
| Tchuente et al. 2004 | sh | 135 | school-aged | 40 mg/kg | 50.4% | 91.4% | yes | ERRgm log-transformed | 88.7% | 0.97 | no |
| Wilkins and Moore 1987 | sh | 33 | school-aged | 40 mg/kg |  | 99.4% | yes | ERRgm log-transformed | 99.4% | 1.00 | yes |
| Wilkins and Moore 1987 | sh | 110 | school-aged | 40 mg/kg |  | 99.8% | yes | ERRgm log-transformed | 99.8% | 1.00 | yes |
| Belizario et al. 2008 | sj | 102 | school-aged | 40 mg/kg | 98.0% | 99.9% | yes | ERRgm unclear | 99.2% | 0.99 | yes |
| Olds et al. 1998 | sj | 203 | school-aged | 40 mg/kg | 94.0% |  |  | na | 98.6% |  | yes |
| Olliaro et al. 2011 | sj | 101 | school-aged | 40 mg/kg | 92.2% | 90.1% | yes | ERRgm log-transformed | 98.3% | 1.09 | yes |
| Barakat and El Morshedy 2011 | sm | 588 | school-aged | 40 mg/kg | 78.8% | 71.2% | no | ERRgm unclear | 95.8% | 1.35 | yes |
| Barakat et al. 2015 | sm | 73 | school-aged | 40 mg/kg | 45.2% |  |  | ERRgm uncured | 87.0% |  | no |
| Berhe et al. 1999 | sm | 541 | school-aged | 40 mg/kg | 83.2% |  |  | na | 96.6% |  | yes |
| Botros et al. 2005 | sm | 32 | school-aged | 40 mg/kg | 62.5% | 63.7% | no | ERRgm unclear | 92.1% | 1.45 | yes |
| Coulibaly et al. 2012 | sm | 35 | preschool | 40 mg/kg | 96.7% | 88.6% | no | ERRgm unclear | 99.0% | 1.12 | yes |
| Degu et al. 2002 | sm | 148 | school-aged | 40 mg/kg | 97.0% | 94.0% |  | ERRgm unclear | 99.1% | 1.05 | yes |
| Erko et al. 2012 | sm | 85 | school-aged | 40 mg/kg | 68.2% | 71.0% | no | ERRgm uncured | 93.5% | 1.32 | yes |
| Erko et al. 2012 | sm | 32 | school-aged | 40 mg/kg | 71.8% | 68.0% | no | ERRgm uncured | 94.3% | 1.39 | yes |
| Erko et al. 2012 | sm | 27 | school-aged | 40 mg/kg | 92.6% | 75.0% | no | ERRgm uncured | 98.3% | 1.31 | yes |
| Friis and Byskov 1989 | sm | 81 | school-aged | 40 mg/kg | 85.2% | 98.0% | yes | ERRgm uncured | 97.0% | 0.99 | yes |
| Garba et al. 2013 | sm | 88 | preschool | syrup pzq40 | 75.0% | 66.7% | no | ERRgm uncured | 95.0% | 1.42 | yes |
| Gryseels et al. 1987 | sm | 176 | school-aged | 40 mg/kg | 78.0% | 99.1% | yes | ERRgm log-transformed | 95.6% | 0.96 | yes |
| Guisse et al. 1997 | sm | 67 | school-aged | 40 mg/kg | 71.0% | 99.0% | yes | ERRgm log-transformed | 94.1% | 0.95 | yes |
| Massoud et al. 1984 | sm | 59 | school-aged | 40 mg/kg | 74.6% |  |  | na | 94.9% |  | yes |
| Metwally et al. 1995 | sm | 69 | school-aged | 40 mg/kg | 61.9% |  |  | na | 92.0% |  | yes |
| Metwally et al. 1995 | sm | 80 | school-aged | 40 mg/kg | 65.4% |  |  | na | 92.8% |  | yes |
| Mohamed et al. 2009 | sm | 46 | school-aged | 40 mg/kg | 100.0% |  |  | na | 99.5% |  | yes |
| Nalugwa et al. 2015 | sm | 149 | preschool | 40 mg/kg | 75.8% |  |  | na | 95.2% |  | yes |
| Nalugwa et al. 2015 | sm | 67 | preschool | 40 mg/kg | 85.1% |  |  | na | 97.0% |  | yes |
| Nalugwa et al. 2015 | sm | 116 | preschool | 40 mg/kg | 86.2% |  |  | na | 97.2% |  | yes |
| Nalugwa et al. 2015 | sm | 25 | preschool | 40 mg/kg | 100.0% |  |  | na | 99.5% |  | yes |
| Navaratnam et al. 2012 | sm | 94 | preschool | syrup pzq40 | 80.9% | 89.0% | no | am | 96.2% | 1.08 | yes |
| Navaratnam et al. 2012 | sm | 109 | preschool | 40 mg/kg | 81.7% | 89.0% | no | am | 96.4% | 1.08 | yes |
| Obonyo et al. 2010 | sm | 101 | school-aged | 40 mg/kg | 65.0% | 84.1% | no | ERRgm unclear | 92.7% | 1.10 | yes |
| Olds et al. 1998 | sm | 82 | school-aged | 40 mg/kg | 56.2% |  |  | na | 90.5% |  | yes |
| Olliaro et al. 2011 | sm | 119 | school-aged | 40 mg/kg | 86.6% | 91.9% | yes | ERRgm log-transformed | 97.3% | 1.06 | yes |
| Selim et al. 2014 | sm | 20 | school-aged | 40 mg/kg | 85.0% |  |  | na | 97.0% |  | yes |
| Simonsen et al. 1990 | sm | 206 | school-aged | 40 mg/kg | 68.0% | 77.0% | no | ERRgm uncured | 93.5% | 1.21 | yes |
| Sousa-Figueiredo et al. 2012 | sm | 305 | preschool | 40 mg/kg | 56.4% | 82.2% | no | am | 90.5% | 1.10 | yes |
| Teesdale et al. 1984 | sm | 18 | school-aged | 40 mg/kg | 50.0% | 92.8% | yes | am | 88.6% | 0.95 | no |
| Thiongo'o et al. 2002 | sm | 173 | school-aged | 40 mg/kg | 67.1% | 67.1% | no | ERRgm log-transformed | 93.2% | 1.39 | yes |
| Thiongo'o et al. 2002 | sm | 91 | school-aged | 40 mg/kg | 77.6% | 99.8% | yes | ERRgm log-transformed | 95.5% | 0.96 | yes |
| Thiongo'o et al. 2002 | sm | 122 | school-aged | 40 mg/kg | 81.1% | 99.9% | yes | ERRgm log-transformed | 96.2% | 0.96 | yes |
| Thiongo'o et al. 2002 | sm | 133 | school-aged | 40 mg/kg | 87.2% | 87.2% | no | ERRgm log-transformed | 97.4% | 1.12 | yes |
| El Tayeb et al. 1988 | sm+sh | 54 | school-aged | 40 mg/kg | 87.7% | 98.0% | yes | ERRgm uncured | 97.5% | 0.99 | yes |
| Kardaman et al. 1985 | sm+sh | 110 | school-aged | 40 mg/kg | 61.8% |  |  | na | 92.0% |  | yes |
| Kardaman et al. 1985 | sm+sh | 101 | school-aged | 40 mg/kg | 66.3% |  |  | na | 93.1% |  | yes |
| Taylor et al. 1988 | sm+sh | 77 | school-aged | 40 mg/kg | 45.5% |  |  | na | 87.1% |  | no |
| Taylor et al. 1988 | sm+sh |  | school-aged | 40 mg/kg | 76.6% |  |  | na | 95.3% |  | yes |

Legend: ERR, egg reduction rate; CR, cure rate; CI, confidence interval; sh, *S. haematobium*; sj, *S. japo*nicum; sm, S. mansoni; gm, geometric mean; am, arithmetic mean;
