# Supplementary figures and images for: Efficacy and safety of praziquantel 40 mg/kg in preschool-aged and school-aged children: a meta-analysis
Source: Parasit Vectors. 2017 Jan 26;10:47. doi: 10.1186/s13071-016-1958-7 (PMC5270314; doi:10.1186/s13071-016-1958-7)

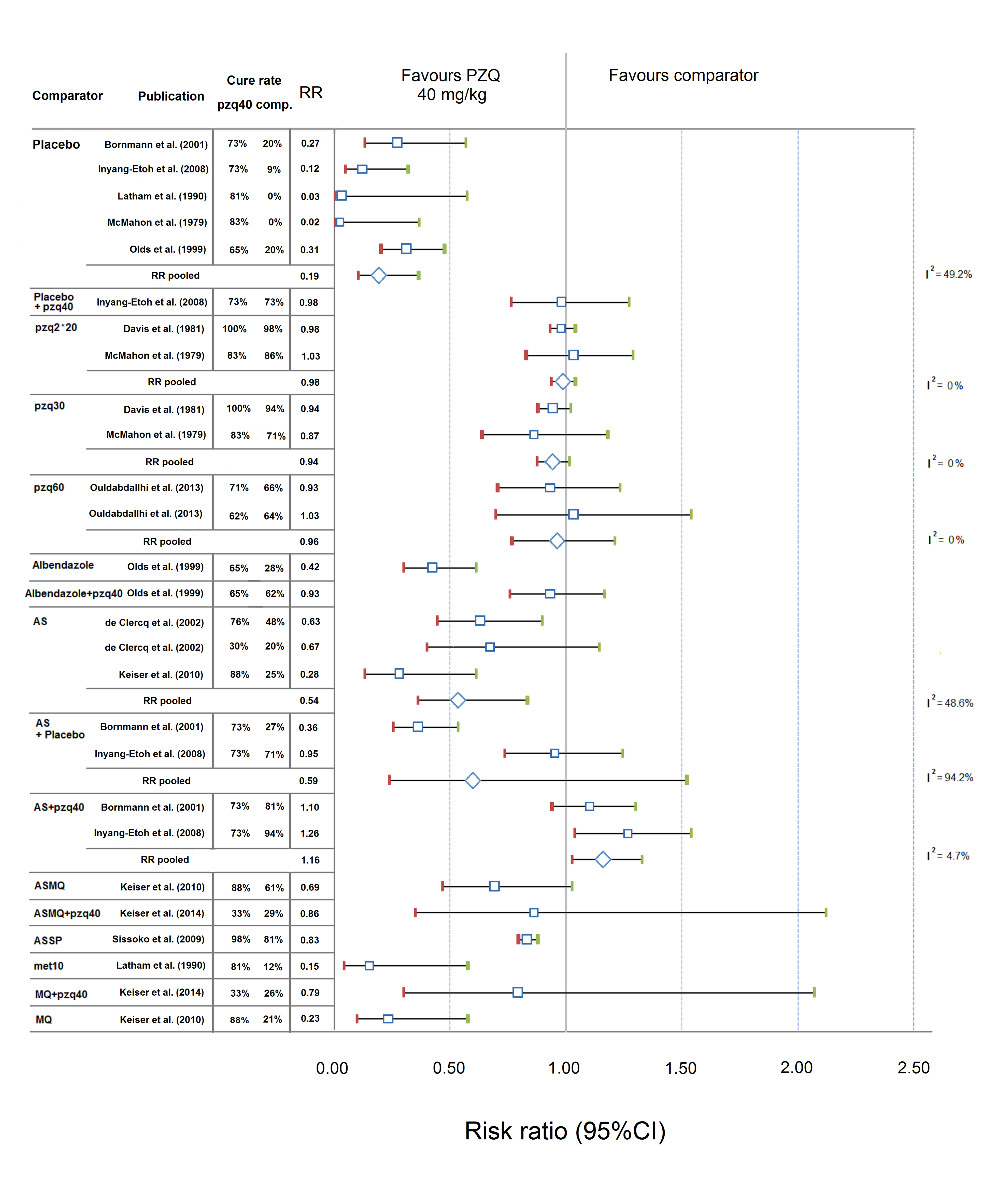

Supplement: Additional file 4: Figure S1. — Forest plot for PZQ 40 mg/kg and comparator treatments for S. haematobium, cure rate (CR) and risk ratio (RR). Abbreviations: pzq40, praziquantel 40 mg/kg; pzq20, praziquantel 20 mg/kg; pzq30, praziquantel 30 mg/kg; pzq60, praziquantel 60 mg/kg; RR, risk ratio; comp, comparator group; AS, artesunate; MQ, mefloquine; ASMQ, artesunate + mefloquine; ASSP, artesunate + sulfadoxine-pyrimethamine; met10, metrifonate 10 mg/kg. (TIF 7292 kb) [file 13071_2016_1958_MOESM4_ESM.tif]

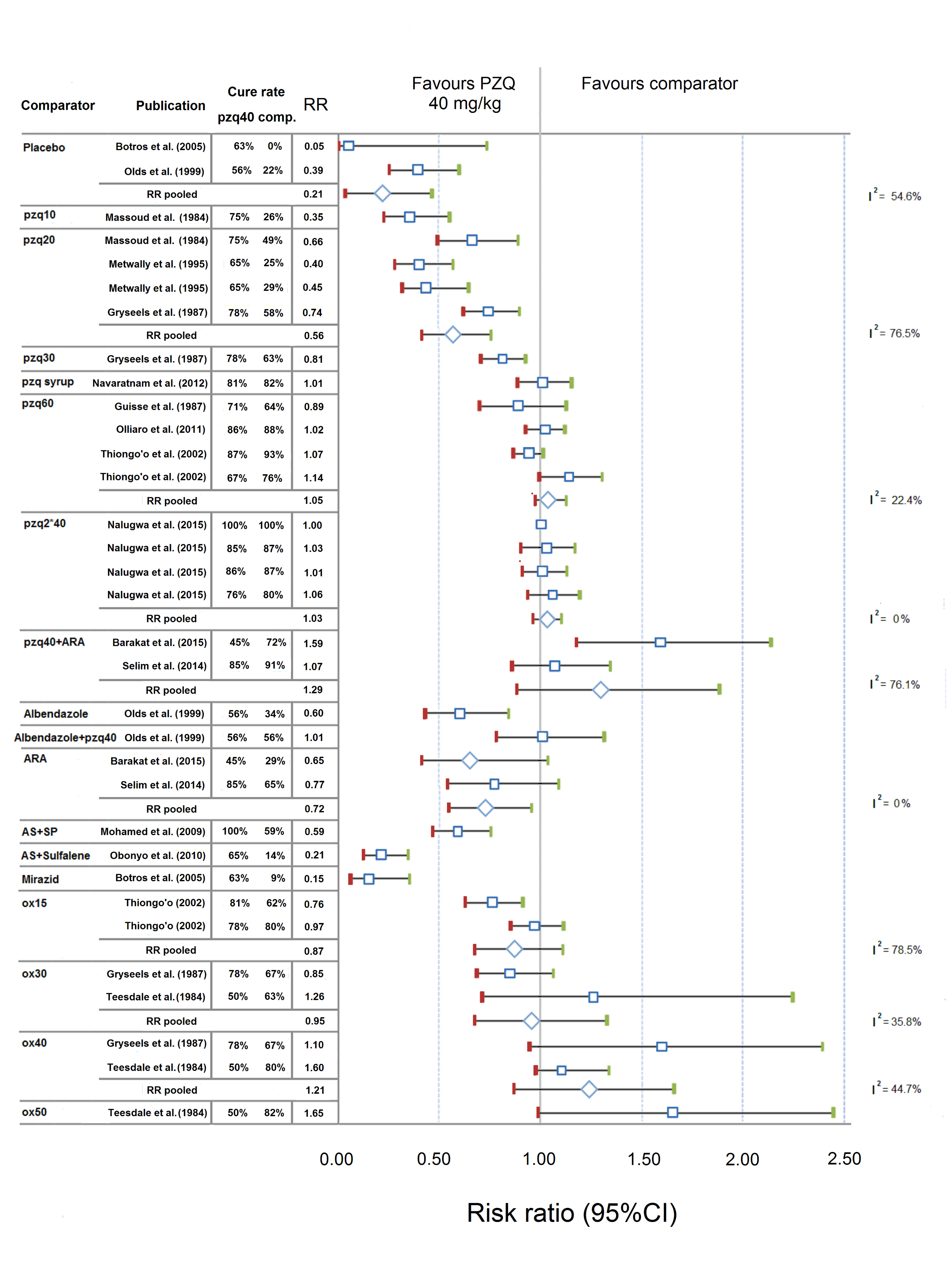

Supplement: Additional file 5: Figure S2. — Forest plot for PZQ 40 mg/kg and comparator treatments for S. mansoni, cure rate (CR) and risk ratio (RR). I2 is calculated for pooled subgroups as = 100%*(Q - df)/Q, where Q is Cochran’s heterogeneity statistic and df the degrees of freedom. Abbreviations: pzq40, praziquantel 40 mg/kg; pzq10, praziquantel 20 mg/kg; pzq20, praziquantel 20 mg/kg; pzq30, praziquantel 30 mg/kg; pzq60, praziquantel 60 mg/kg; pzq syrup, praziquantel syrup 40 mg/kg; ARA arachidonic acid; ox, oxamniquine; RR, risk ratio; comp, comparator group; I2, Higgins’ I squared. (TIF 8364 kb) [file 13071_2016_1958_MOESM5_ESM.tif]
